# Improving pediatric TB diagnosis in North Kivu (DR Congo), focusing on a clinical algorithm including targeted Xpert MTB/RIF on gastric aspirates

**DOI:** 10.1186/s13031-020-00281-1

**Published:** 2020-05-14

**Authors:** Daan Van Brusselen, Erica Simons, Tony Luendo, Delphine Habarugira, Jimmy Ngowa, Nadine Neema Mitutso, Zakari Moluh, Mieke Steenssens, Rachelle Seguin, Hilde Vochten, Lucien Ngabo, Petros Isaakidis, Gabriella Ferlazzo

**Affiliations:** 1 Médecins Sans Frontières (MSF), Operational Center Brussels, Rue de l’Arbre-Bénit 46, 1050 Brussels, Belgium; 2 Médecins Sans Frontières (MSF), Mission RDC, 11 Avenue Massamba, Quartier Bassoko, Ngaliema, Kinshasa, Democratic Republic of the Congo; 3 Department of (Tropical) Pediatrics, GZA Hospitals, Oosterveldlaan, 22 Antwerp, Belgium; 4 Department of Public Health and Primary Care, Faculty of Medicine, Ghent University, Corneel Heymanslaan 10, entrance 42 (building K3), 4th floor, Ghent, Belgium; 5 Bureau Central de Zone (de Santé), Masisi, Ministry of Health of North Kivu, Hôpital Régional de Référence de Masisi, Zone de Santé de Masisi, Nord-Kivu, Democratic Republic of the Congo; 6 Médecins Sans Frontières, Southern African Medical Unit (SAMU), Médecins Sans Frontières, Zurich House, 7th Floor, 70 Fox Street, Marshalltown, Johannesburg, South Africa

**Keywords:** Operational research, Tuberculosis (TB), Pediatric, Diagnosis, Congo, Xpert MTB/RIF, Gastric aspirates

## Abstract

**Background:**

The incidence of tuberculosis (TB) in the Democratic Republic of the Congo (DRC) is 323/100,000. A context of civil conflict, internally displaced people and mining activities suggests a higher regional TB incidence in North Kivu. Médecins Sans Frontières (MSF) supports the General Reference Hospital of Masisi, North Kivu, covering a population of 520,000, with an elevated rate of pediatric malnutrition. In July 2017, an adapted MSF pediatric TB diagnostic algorithm, including Xpert MTB/RIF on gastric aspirates (GAs), was implemented. The aim of this study was to evaluate whether the introduction of this clinical pediatric TB diagnostic algorithm influenced the number of children started on TB treatment.

**Methods:**

We performed a retrospective analysis of pediatric TB cases started on treatment in the inpatient therapeutic feeding centre (ITFC) and the pediatric ward. We compared data collected in the second half (July to December) of 2016 (before introduction of the new diagnostic algorithm) and the second half of 2017. For the outcome variables the difference between the two years was calculated by a Pearson Chi-square test.

**Results:**

In 2017, 94 GAs were performed, compared to none in 2016. Twelve percent (11/94) of samples were Xpert MTB/RIF positive. Sixty-eight children (2.9% of total exits) aged between 3 months and 15 years started TB treatment in 2017, compared to 19 (1.4% of total exits) in 2016 (p 0.002). The largest increase in pediatric TB diagnoses in 2017 occurred in patients with a negative Xpert MTB/RIF result, but clinically highly suggestive of TB according to the newly introduced diagnostic algorithm. Fifty-two (3.1%) children under five years old started treatment in 2017, as compared to 14 (1.3%) in 2016 (p 0.004). The increase was less pronounced and not statistically significant in older patients: sixteen children (2.6%) above 5 years old started TB treatment in 2017 as compared to five (1.3%) in 2016 (p 0.17).

**Conclusion:**

After the introduction of an adapted clinical pediatric TB diagnostic algorithm, including Xpert MTB/RIF on gastric aspirates, we observed a significant increase in the number of children – especially under 5 years old – started on TB treatment, mostly on clinical grounds. Increased ‘clinician awareness’ of pediatric TB likely played an important role.

## Background

The UN Sustainable Development Goals have prioritized ending the epidemic of tuberculosis (TB) by 2030 [1]. This goal is far from being realized. TB remains the leading infectious killer globally. In 2018, there were 10.0 million estimated new TB cases, with over 1.4 million deaths [1]. Ten to 15% of the TB patients in low-income countries (LIC) are estimated to be children < 15 years [2]. Less than half (43%) of the estimated 1 million children with TB were reported to national TB programs, indicating massive under-diagnosis and insufficient access to appropriate care [1, 3].

The estimated incidence of TB in the Democratic Republic of the Congo (DRC) is 323/100.000 according to WHO [4]. The real TB burden in North Kivu – a province in Eastern Congo – is unknown, but a context of civil conflict, displaced people and mining activities suggests a higher regional incidence. The Masisi health zone – in the North Kivu province – covers a population of 520,000. The estimated number of people with TB in the Masisi health zone – as reported by the Ministry of Health (MoH) – has grown in the last couple years: from 310 patients in 2015 (60/100.000), to 594 (114/100.000) in 2016 and 605 in 2017 (116/100.000), but massive underreporting is suspected [5]. Twentyfive percent of the TB patients are children. HIV prevalence in the general population of the region is less than 1%.

The top 4 of most frequent pathologies in children under 5 years old in Masisi are lower respiratory tract infections as the first, followed by severe malaria, sepsis and non-bloody diarrhea. The burden of malnutrition is high, as shown by a nutrition survey conducted in 2016 by MoH which reported a prevalence of global acute malnutrition (GAM) of 4,1% (CI 2,8 – 5,9) and severe acute malnutrition (SAM) of 1% (CI 0,5% - 2,3%) respectively for children of 0–59 months in the Masisi region [5]; this is most likely an underestimation because of the highly volatile situation in North Kivu. Undernutrition increases the risk of TB and TB can cause or worsen undernutrition [6]. One study estimated that 26% of overall TB cases in 22 high-burden countries are attributable to undernutrition [7].

Even if pulmonary TB (PTB) is still the most frequent presentation of TB in children, the burden of extrapulmonary TB (EPTB) is certainly higher than in adults [8, 9]. It is hard to get good samples in EPTB, but PTB is also paucibacillary, resulting in the fact that bacteriologic confirmation is achievable in less than 50% of all children with TB [10]. Therefore – even with the most recent advances in molecular diagnostics – TB in children remains mostly a clinical diagnosis.

Because of their low sensitivity and specificity in children < 5 years old, malnourished and HIV+ children, and the fact that Masisi is a high prevalence area, tuberculin skin test (TST) or interferon gamma release assay (IGRA) are of limited use in our setting [10, 11]. Chest X-ray can be a useful tool for diagnosis of pulmonary TB in children, but is not very sensitive and specific, and operator-dependent [12]. Obtaining expectorated sputum without induction from children for detection of acid fast bacilli (AFB) is difficult and its examination is of low yield [13]. The Xpert MTB/RIF, an automated nucleic acid amplification test that can simultaneously identify *M. tuberculosis* and detect rifampicin resistance, is (although less sensitive than cultures) very specific, easier to implement than cultures in LIC and less time-consuming [14]. The sensitivity and specificity of Xpert MTB/RIF were found to be quite similar for gastric aspiration (GA) and induced sputum [15]. Because the implementation of diagnostic GA is easier in LIC, we have chosen to include Xpert MTB/RIF on GA in our pediatric diagnostic algorithm.

The aim of this study is to evaluate whether the introduction of the Médecins Sans Frontières (MSF) pediatric TB diagnostic algorithm – which included targeted Xpert MTB/RIF on GA samples – influenced the number of children started on TB treatment.

## Methods

### Study design and study population

We performed a retrospective analysis of TB diagnosis, TB treatment and overall mortality in children aged between 28 days of life and 18 years old admitted to the general pediatric ward and the inpatient therapeutic feeding centre (ITFC) in the General Reference Hospital (GRH) of Masisi.

### MSF program description

Since 2007, MSF has supported the Masisi health zone. The aim is to provide free high-quality medical care to a population that has experienced nearly constant conflict and violence over the past 25 years. At the start, support was given only to the surgical service (especially for war-wounded), to victims of sexual violence and internally displaced persons (IDPs) through mobile clinics. Step by step, this support was extended to all services of the Masisi GRH, offering 220 beds and providing care for more than 17,000 people (among them approximately 35% children) in 2017, to the Masisi health center (HC) and to the Nyabiondo referral health center, with inpatient department (IPD) capacity, 20 km away from Masisi. The latter centers refer children to the GRH in case of complications, including when TB is suspected. Ambulances connect the supported structures. All services are free of charge.

### Diagnostic methods and operational definitions

**Fig. 1:**
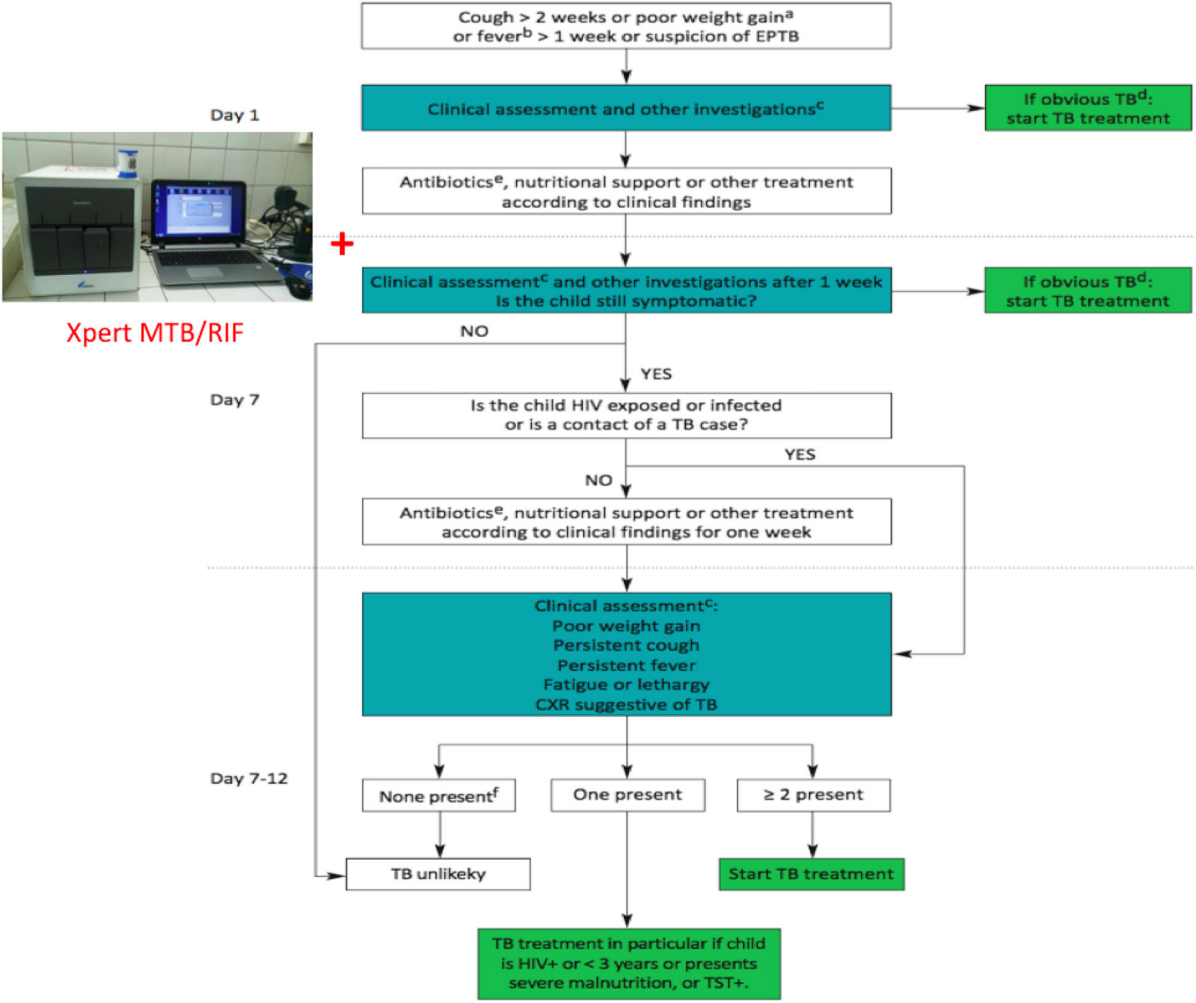
MSF Pediatric TB Diagnostic Algorithm. Médecins Sans Frontières and Partners In Health. Tuberculosis: Practical guide for clinicians, nurses, laboratory technicians and medical auxiliaries. 2014 Edition

We introduced an adapted MSF pediatric TB diagnostic algorithm – including Xpert MTB/RIF on GA samples – on July 1st 2017 because we had the impression that clinicians underdiagnosed pediatric TB. Gastric aspirates were performed in a targeted way, following the MSF pediatric TB diagnostic algorithm (Fig. 1) for all children admitted in pediatrics or ITFC with a suspicion of TB. All children with cough > 2 weeks, poor weight gain on therapeutic feeding (if malnourished), fever > 1 week or a suspicion of EPTB, were assessed clinically and investigations (e.g. chest x-ray in case of suspicion of pulmonary TB) were done according to the symptoms (Fig. 1). Antibiotics (according to the clinical diagnosis) were given for one week, as well as nutritional support and other treatment according to the clinical findings. If after one week there was no improvement of the symptoms or nutritional status, another clinical assessment was done as well as Xpert MTB/RIF on a GA sample (Fig. 1). Gastric aspiration (GA) was performed according to the MSF TB Guideline [16]. After correctly placing the nasogastric catheter, first the gastric fluid present in the stomach was aspirated and placed in the sputum container, then the stomach was rinsed with 20 to 30 ml of sterile water / NaCl 0,9%, after which another episode of suctioning was performed. The additionally suctioned fluid was added to the first sample [16]. Samples were immediately sent to the local lab (in GRH), where they were analyzed with our Xpert MTB/RIF. Xpert results were given the same day or the day after sample collection. The child was started on TB treatment if the result of the Xpert was positive, but also in case of a strong clinical suspicion of TB (‘obvious TB’) (Fig. 1). In case of a negative Xpert result and no ‘obvious TB’, but persisting symptoms in an HIV-negative child, another week of symptomatic treatment and nutritional support (in case of malnutrition) was given. If the final clinical assessment contained two or more of the following elements, TB treatment was started anyway: poor weight gain, peristent cough, persistent fever, fatigue/lethargy, chest x-ray suggestive of TB (Fig. 1). If the child was HIV-exposed (if very young) or – positive, or a contact of a TB patient, no additional week of antibiotics or nutritional support was given. In this case the final clinical assessment to decide whether the child was started on TB treatment or not, was already done after the first week of complete treatment (Fig. 1).

### Data collection and analysis

We used routinely collected data from the MSF monthly medical report, the pediatric TB register and the lab Xpert MTB/RIF register. All patient data were anonymized. We compared the 2nd half of 2016 with the 2nd half of 2017. Categorical variables are presented as n (%) and their *p*-values were calculated by χ^2^. Only for median ages a non-parametric Mann-Whitney test was used. Descriptive statistics were performed in SPSS (version 24.0; SPSS Inc., IBM, Chicago, IL USA).

### Ethical approval and consent to participate

This study satisfies the criteria for reports using routinely collected programmatic data set by the Médecins Sans Frontières Ethics Review Board (ERB). Patient identifying information was removed prior to analysis. As this was a study of routinely collected monitoring data, patient consent was not required.

## Results

In the last semester of 2017 the total number of exits from the pediatric department was 1546 as compared to 869 in the last semester of 2016. The number of exits from ITFC was 762 as compared to 552 in 2016 (Table 1). The increase in both departments was partly related to an increase in the number of malaria cases (968 in 2017 vs. 839 in 2016) and the increase in the ITFC was partly related to ruptures of therapeutic feeding in non-MSF supported structures. However, the main increase in both departments was related to a higher number of displaced people. The top 4 of most frequent pathologies in pediatrics has been constant.

**Table 1:** General information and overall mortality in the pediatric ward and ITFC of Masisi GRH. Categorical variables are presented as n (%) and their *p*-values were calculated by χ^2^

Column1	2nd semester 2016	2nd semester 2017	***p***-value
**Total exits**	1421	2308	N/A
**Overall inpatient pediatric mortality** (% of all children discharged from both wards)	60 (4%)	59 (3%)	0.005
**Mortality ITFC** (% of children discharged from ITFC)	43 (8%)	46 (6%)	0.21
**Mortality pediatric ward** (% of children discharged from pediatric ward)	17 (2%)	13 (1%)	0.02

In 2017, 94 GAs were performed according to the MSF pediatric TB diagnostic algorithm, including Xpert MTB/RIF on GA samples (Fig. 1), compared to none in 2016. All GA samples were tested with Xpert MTB/RIF and 12% (11/94) were Xpert MTB positive. No resistance to rifampicin was detected among the 11 positive Xpert samples (Table 1). Sixty-eight children (2.9% of all children discharged from either ‘general pediatrics’ or ITFC) between 3 months and 15 years started TB treatment in the second half of 2017, compared to 19 (1.4% of all children discharged) in 2016 (p 0.002) (Table 2). When stratified by age, 52 (3.1%) children under five years old started treatment in the second half of 2017, as compared to 14 (1.3%) in the second half of 2016 (p 0.004) (Table 2). The increase was less pronounced and not statistically significant in older patients: sixteen children (2.6%) above 5 years old started TB treatment in the second half of 2017 as compared to five (1.3%) in the second half of 2016 (p 0.17) (Table 2). Fifty-seven patients with a negative Xpert MTB/RIF result, but clinico-radiological findings highly suggestive of TB – as defined in the TB diagnostic algorithm, started treatment based on clinical criteria in the second half of 2017 (Fig. 1). Five of the pediatric TB patients (26%) were HIV-positive in 2016 as compared to 2 (3%) in 2017 (Table 2).

**Table 2:** Specific information related to pediatric TB patients in GRH Masisi. Categorical variables are presented as n (%) and their p-values were calculated by χ^2^. Only for median ages a non-parametric Mann-Whitney test was used. N/A = not applicable

Column1	2nd semester 2016	2nd semester 2017	***p***-value
**Total pediatric TB diagnoses** (% of all children discharged)	19 (1.4%)	68 (2.9%)	0.002
**TB diagnoses under 5 years** (% of all children under 5 discharged)	14 (1.3%)	52 (3.1%)	0.004
**TB diagnoses 5 to 18 years** (% of all children above 5 discharged)	5 (1.3%)	16 (2.6%)	0.17
**GA Xpert MTB positive** (% of all pediatric TB diagnoses)	No Xpert done	11 (16%)	N/A
**Rifampicine resistence detected** (% of all pediatric TB diagnoses)	No Xpert done	0 (0%)	N/A
**HIV-positive** (% of all pediatric TB diagnoses)	5 (26%)	2 (3%)	0.07
**Median Age** (Interquartile Range)	20 m (12 m – 62 m)	36 m (23,5 m – 60 m)	0.27
**Female** (% of all pediatric TB diagnoses)	6 (32%)	36 (53%)	0.10
**Pediatric TB fatality rate** (% of all pediatric TB diagnoses)	8 (42%)	17 (25%)	0.30
**TB deaths in ITFC** (% of all deaths in ITFC)	5 (12%)	16 (35%)	0.01
**TB deaths in pediatric ward** (% of all deaths in pediatric ward)	3 (18%)	1 (7%)	0.43

The total number of deaths in the pediatric ward was 17 (2% of all exits in this ward) in the last semester of 2016 versus 13 (1%) in 2017 (p 0.02); at ITFC the number of deaths was 43 (8% of all exits in this ward) versus 46 (6%) (p 0.21), respectively (Table 1). The TB fatality rate went from 42% of pediatric TB patients (*n* = 8) in 2016 to 25% (*n* = 17) in 2017 (p 0.3) (Table 2). In 2017 one child (7% of all deaths in the department) who died in pediatrics and 16 (35%) of children who died in ITFC had a diagnosis of TB, as compared to three (18%) (p 0.43) and five (12%) (p 0.01) in 2016 (Table 2).

## Discussion

The aim of this study was to evaluate whether the introduction of a clinical pediatric TB diagnostic algorithm, including Xpert MTB/RIF on GA samples, influenced the number of children started on TB treatment. We have demonstrated a significant increase in the number of children started on medication after the introduction of this algorithm. This increase was clearly statistically significant in children under 5 years old, while local clinicians did before not typically consider TB to be prevalent in this age group.

Progress in TB diagnosis in children, who have only recently been recognized to have a huge burden of TB disease, has been minimal. Over 96% of children who die from TB worldwide never access treatment, especially in LIC and conflict-torn regions [17]. Furthermore less than 15% of eligible children at high risk of developing TB receive preventive therapy [1, 18].

As stated in the introduction, pediatric TB diagnoses are primarily clinically-based [10, 18, 19]. The clinical algorithm developed for LIC by the MSF pediatric working group, together with the Southern African Medical Unit (SAMU), can be used as a tool for well-nourished and malnourished children (Fig. 1). Because of the known role of TB as comorbidity among children with acute malnutrition, the MSF pediatric TB diagnostic algorithm emphasizes the need to routinely screen all malnourished children for TB and manage those at risk appropriately (Fig. 1). Asking about contacts with TB patients should be included as part of the initial history for children in nutrition programs, as outlined in the WHO manual on the management of severe malnutrition, followed by a diagnostic algorithm, like ours [20, 21] (Fig. 1).

### Gastric aspiration (GA) and Xpert MTB/RIF

Zar et al stated in 2005 that sputum induction is better than GA in children because three GAs were necessary to obtain the same diagnostic yield as one induced sputum specimen in her setting in Cape Town and the risk of nosocomial transmission is lower in children than in adults [13]. But sputum induction should be done in an isolation room with adequate ventilation and proper personal respiratory protection, which is not always easy in low-resource settings [22]. Newer studies found that the bacteriological yield of GA and sputum induction are more or less similar [15, 23]. For these reasons and because of its simplicity, GA remains the most common method for obtaining respiratory samples from children. Therefore and in order to increase not only the sensitivity, but especially awareness of clinicians about pediatric TB, we decided to include Xpert MTB/RIF on GA in our protocol.

It is important that the procedure of gastric aspiration is performed correctly to increase the diagnostic yield. In many settings aspiration is only done once, while it is advised to rinse the stomach with sterile water/NaCl 0,9% and to suction again afterwards, especially when not a lot of sputum was obtained upon the first suctioning [16, 24]. At the time of the introduction of the Xpert MTB/RIF in Masisi, the MSF TB Protocol for gastric aspiration was reinforced by theoretical and bedside teaching, with successful results. Use of the Xpert MTB/RIF test on GA may be especially beneficial in settings where mycobacterial culture is not feasible, like LIC and conflict zones, esp. for children who cannot produce sputum spontaneously.

Although age-disaggregated data on MDR-TB are not even reported to national authorities, indications are that less than 10% of the estimated 30.000 children who develop MDR-TB every year are diagnosed [18, 25, 26]. The fact that Xpert MTB/RIF already comes with an initial (and quick) evaluation of rifampicin resistance (which should be further explored by culture if positive) is another advantage of the use of Xpert MTB/RIF on GA samples as a part of our algorithm. Fortunately we did not find any cases of rifampicin resistance in our Xpert positive pediatric cases, but the sample size was very low.

### Clinical diagnosis

We observed a significant increase in the number of children started on TB treatment after implementation of our clinical algorithm. Most of the children who started treatment were not positive on Xpert MTB/RIF, which raises questions about its necessity in our algorithm. However, the high incidence of TB in children is often seriously underestimated. This was also the case with our local clinicians in Masisi GRH in DRC. The doctors were surprised that several pediatric samples were positive, which brought about a behaviour change and lowered the threshold to start TB treatment on clinical grounds as well (using our newly introduced clinical diagnostic algorithm). We believe that increased ‘clinician awareness’ (becoming more aware that children do frequently have TB) played an important role in the higher number of diagnoses in 2017.

The overall pediatric TB fatality rate went down from 42 to 25% after the introduction of our algorithm (Table 2), what could point in the direction of earlier diagnoses, but this difference was not statistically significant. Thirty-five percent of children who died in our ITFC had a diagnosis of TB in 2017 (almost 3 times more than in 2016): this might be an overestimation, since TB treatment can sometimes be a ‘treatment of last resort’ (especially now that Masisi doctors are more aware of TB as a frequent pediatric diagnosis) (Table 2). But a high number of TB deaths in the malnutrition ward does correspond to the literature, and lowering the threshold for TB treatment in these very sick malnourished children who mostly have several infections together however, is justified.

Besides the implementation of our TB diagnostic algorithm, different more general pediatric trainings on diverse subjects (pediatric resuscitation, malaria diagnosis and treatment, shock management and rational use of antibiotics) were also given in 2017 and the reduced overall mortality in the general pediatric ward can of course not be solely attributed to our intervention; although, it could be one of the contributing factors. The significant increase in the number of children started on TB treatment, however, is, in our view, mostly due to the newly introduced adapted clinical pediatric TB diagnostic algorithm.

### Limitations

This study has several limitations, including the fact that it is a retrospective analysis of routinely collected data, in an unstable setting. Therefore, some important patient and disease information was missing. Our main data source was the pediatric TB register, where the site of the disease (PTB or EPTB) or the ward of admission (pediatric or ITFC) was not systematically recorded. Another limitation is the lack of a gold standard to confirm TB diagnosis among children started on TB treatment on clinical/radiological basis. We could therefore not assess the accuracy of the diagnostic algorithm and we cannot confirm that all children who were diagnosed and started on treatment actually had TB.

## Conclusion

Great progress has been made in the ‘End-TB’ strategy of WHO in recent years. However, pediatric TB still remains underdiagnosed and an important cause of under-five mortality, especially in malnourished children [27, 28].

Pediatric TB is still a clinical diagnosis. In Masisi we have shown that improving TB diagnosis in children - with an adapted clinical TB diagnostic algorithm, including Xpert MTB/RIF on GA samples - can be feasible and effective in a Central-African conflict setting with a high malnutrition and TB burden. Increased ‘clinician awareness’ of pediatric TB likely played an important role in this.

## Data Availability

The datasets generated and/or analyzed during the current study are not publicly available due to the fact that it concerns patient data from the Congolese Ministry of Health, but are (without patient data) available from the corresponding author on reasonable request.

## Abbreviations

AFB: Acid fast bacilli
DRC: Democratic Republic of Congo
GA: Gastric aspirate
GAM: Global acute malnutrition
HGR: Hôpital général de référence
HIV: Human immunodeficiency virus
ITFC: Inpatient Therapeutic Feeding Centre
IGRA: Interferon gamma release assay
IPT: Isoniazid preventive therapy
LIC: Low-income countries
LTBI: Latent tuberculosis infection
MDR: Multidrug resistant
MSF: Médecins Sans Frontières (Doctors Without Borders)
MoH: Ministry of health
SAM: Severe acute malnutrition
SAMU: Southern African Medical Unit
TB: Tuberculosis
TST: Tuberculin skin test
Xpert MTB/RIF: molecular test for *Mycobacterium tuberculosis*, including Rifampicin resistance test
